# Three-dimensional cell shapes and arrangements in human sweat glands as revealed by whole-mount immunostaining

**DOI:** 10.1371/journal.pone.0178709

**Published:** 2017-06-21

**Authors:** Ryuichiro Kurata, Sugiko Futaki, Itsuko Nakano, Fumitaka Fujita, Atsushi Tanemura, Hiroyuki Murota, Ichiro Katayama, Fumihiro Okada, Kiyotoshi Sekiguchi

**Affiliations:** 1 Laboratory of Advanced Cosmetic Science, Graduate School of Pharmaceutical Sciences, Suita, Osaka University, Osaka, Japan; 2 Fundamental Research Institute, Mandom Corporation, Osaka-city, Osaka, Japan; 3 Division of Matrixome Research and Application, Institute for Protein Research, Osaka University, Suita, Osaka, Japan; 4 Department of Dermatology, Graduate School of Medicine, Osaka University, Suita, Osaka, Japan; University of Toronto, CANADA

## Abstract

Because sweat secretion is facilitated by mechanical contraction of sweat gland structures, understanding their structure-function relationship could lead to more effective treatments for patients with sweat gland disorders such as heat stroke. Conventional histological studies have shown that sweat glands are three-dimensionally coiled tubular structures consisting of ducts and secretory portions, although their detailed structural anatomy remains unclear. To better understand the details of the three-dimensional (3D) coiled structures of sweat glands, a whole-mount staining method was employed to visualize 3D coiled gland structures with sweat gland markers for ductal luminal, ductal basal, secretory luminal, and myoepithelial cells. Imaging the 3D coiled gland structures demonstrated that the ducts and secretory portions were comprised of distinct tubular structures. Ductal tubules were occasionally bent, while secretory tubules were frequently bent and formed a self-entangled coiled structure. Whole-mount staining of complex coiled gland structures also revealed the detailed 3D cellular arrangements in the individual sweat gland compartments. Ducts were composed of regularly arranged cuboidal shaped cells, while secretory portions were surrounded by myoepithelial cells longitudinally elongated along entangled secretory tubules. Whole-mount staining was also used to visualize the spatial arrangement of blood vessels and nerve fibers, both of which facilitate sweat secretion. The blood vessels ran longitudinally parallel to the sweat gland tubules, while nerve fibers wrapped around secretory tubules, but not ductal tubules. Taken together, whole-mount staining of sweat glands revealed the 3D cell shapes and arrangements of complex coiled gland structures and provides insights into the mechanical contraction of coiled gland structures during sweat secretion.

## Introduction

Understanding the mechanisms of exocrine gland secretion is of clinical importance because defective secretion can markedly reduce the quality of life of patients suffering from disorders such as hyperhidrosis and dry mouth [1, 2]. Because mechanical stress facilitates gland secretion (e.g. contraction of myoepithelial cells surrounding secretory portions), structural dissection of exocrine mammary and salivary glands has been performed by conventional histological methods. The mammary gland is an alveolar gland containing enlarged secretory acini with large lumina filled with milk [3]. Contractions of the stellate-shaped myoepithelial cells around secretory alveoli eject milk from the mammary glands. Salivary glands are typical tubuloalveolar exocrine glands containing secretory alveolar and tubular elements [4, 5]. The stellate-shaped myoepithelial cells surround the secretory portions and some parts of ducts in salivary glands, expelling saliva from acini.

Sweat glands have also been schematically delineated by histological analyses. Sweat glands are unbranched coiled tubules consisting mainly of secretory portions and ducts, the latter comprising epidermal, straight, and coiled ducts. The secretory portions consist of secretory luminal cells and encompassing myoepithelial cells, while the ducts consist of luminal and basal cells. Myoepithelial cells are believed to modulate sweating through contraction of secretory portions [2, 6]. The three-dimensional (3D) coiled structures of sweat glands have been assessed histologically. However, conventional histological methods are limited in determining complex 3D structures of organs, because information is lost during sample processing. Whole-mount staining methods have been developed to obtain detailed anatomical information about complex 3D organs such as the brain [7–11]. Recently, whole-mount staining of mammary glands has allowed visualization of the 3D structures of duct and secretory portions [12], indicating that whole-mount staining is a powerful tool that might determine the detailed 3D structures of sweat gland coiled regions.

In this study, whole-mount analyses revealed the detailed 3D structures of sweat glands. Their secretory tubules were found to form a self-entangled coiled structure. Myoepithelial cells of secretory portions were highly elongated and arranged longitudinally in the entangled secretory portion, suggesting that myoepithelial cells align in spatially characteristic patterns on the secretory portions to effectively expel sweat from the tubular structures. These findings provide insights into the cellular mechanisms that govern sweat gland activity and may lead to clinical benefits for patients with impaired thermoregulation.

## Materials and methods

### Human skin tissues

Fresh human skin tissues were obtained with informed consent from Osaka University Hospital (Osaka, Japan), Kinugasa Clinic (Osaka, Japan), and Mediclude (Tokyo, Japan). Experiments using human skin were approved by the Ethics Committee of Osaka University.

### Antibodies

Primary antibodies used for immunohistochemical, immunofluorescence, and whole-mount staining included anti-keratin 8 (K8; Progen, Heidelberg, Germany), anti-α-smooth muscle actin (αSMA; Abcam, Cambridge, MA), anti-keratin 77 (K77; Abcam), anti-S100 calcium binding protein A2 (S100A2; Novus Biologicals), anti-CD29 (Abcam), anti-CD49f (Millipore, Milford, MA), anti-platelet endothelial cell adhesion molecule (CD31; Abcam), and anti-protein gene product 9.5 (PGP9.5; Abcam). Secondary antibodies were species-specific horseradish peroxidase (HRP)-conjugated secondary antibodies (Abcam) and species-specific fluorescent dye-conjugated secondary antibodies (Invitrogen, Carlsbad, CA). F-actin was stained with Alexa Fluor 488 and 594 Phalloidin (Invitrogen).

### Immunohistochemical analysis

Human skin tissues were embedded in OCT compound (Sakura Finetechnical Co., Tokyo, Japan) and frozen in liquid nitrogen-chilled 2-methylbutane. Cryosections were prepared, fixed in 4% formaldehyde/phosphate-buffered saline (PBS), cold methanol, or cold acetone, and blocked with 1% goat serum (Dako, Carpinteria, CA) or 3% bovine serum albumin (BSA) (Sigma, St. Louis, MO) in PBS, followed by overnight incubation with primary antibodies at 4°C. After three PBS washes, sections were treated with HRP-conjugated secondary antibodies, followed by color development using 3,3-diaminobenzidine. After counterstaining with hematoxylin, the sections were examined under a DM2500 microscope (Leica, Wetzlar, Germany). Hematoxylin and eosin (HE) staining was performed by conventional methods. For double immunofluorescence staining, sections were prepared, fixed in 4% formaldehyde/PBS, cold methanol, or cold acetone, and then incubated overnight with primary antibodies at 4°C. After washing in PBS, the sections were treated with secondary antibodies for 1 h, washed with PBS, and then stained with Hoechst 33342 to visualize nuclei. All procedures were performed at room temperature. Immunofluorescence images were recorded under an FV1200 confocal laser-scanning microscope (Olympus, Japan).

### Whole-mount sweat gland preparation

For each human skin tissue sample, connective tissues encasing sweat gland secretory regions were dissected from the subcutaneous tissue using fine forceps. The resulting skin tissues were vertically cut into small pieces with sharp scissors. Sweat glands in the dermis were visualized using 10 μM neutral red (Sigma) in PBS and collected using fine forceps [13]. The isolated sweat glands were fixed for 30 min with 4% formaldehyde in PBS, washed three times (1 h/wash) with 2% BSA/PBTT (0.1% Tween-20 and 0.1% Triton-X 100 in PBS), and then incubated overnight at 4°C with primary antibodies in 2% BSA/PBTT. The sweat glands were washed and incubated overnight at 4°C with secondary antibodies plus Hoechst 33342 (Molecular Probes, Eugene, OR) and phalloidin (Invitrogen) in 2% BSA/PBTT. The following day, the tissues were washed and then incubated overnight in 80% glycerol prior to dissection for 3D imaging. Fluorescence images of whole sweat glands were acquired under the FV1200 confocal laser-scanning microscope using 405, 473, 559, and 635 nm lasers and an FV12 high-sensitivity detector with 10× [UPlanSApo, numerical aperture (NA) = 0.40, working distance = 3.1 mm], 20× (UPlanSApo, NA = 0.75, working distance = 0.6 mm), or 40× (UPlanSApo, NA = 0.95, working distance = 0.18 mm) objective lenses.

## Results

### Molecular profiling of human sweat gland compartments

**Fig 1:**
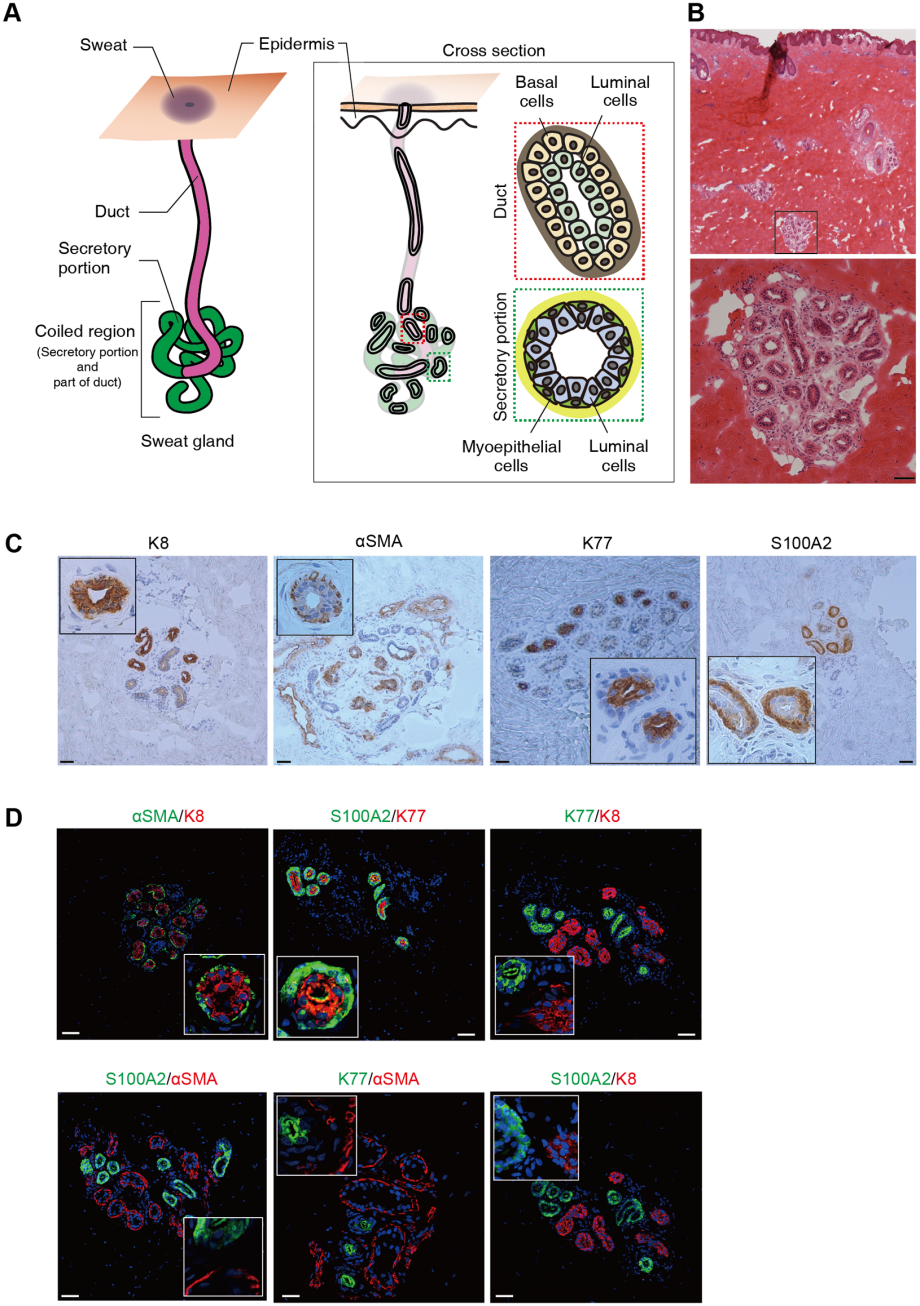
Molecular profiling of human sweat gland compartments. (A) Schematic illustration of a human sweat gland and its main tissue segments. (Left) Diagram of the sweat gland structure in human skin; (center) diagram of a sweat gland cross section in human skin; (upper right) diagram of the cellular arrangement in the duct consisting of luminal and basal cells surrounded by basement membrane; (lower right) diagram of the cellular arrangement in the secretory portion consisting of luminal and myoepithelial cells surrounded by basement membrane. (B) Cross section of sweat gland coiled tubules in the deep dermis of human skin. HE-stained skin cross section shows the sweat glands surrounded by adipocytes and connective tissue. The coiled regions of sweat glands were detected as rounded structures in the hypodermis. Boxed area in the upper panel is magnified in the lower panel. (C) Expression patterns of sweat gland cell markers K8, αSMA, K77, and S100A2 in human sweat glands. K8 and K77 were expressed in parts of the luminal cell layers, and αSMA and S100A2 in parts of the basal cell layers. Insets show magnified views of the sweat glands. (D) Double immunofluorescence detection of sweat gland markers K8, αSMA, K77, and S100A2 in sweat glands. Insets show magnified views. Nuclei (blue) were counterstained with Hoechst 33342. (B–D) Scale bars: 50 μm.

Sweat gland coiled structures consist of secretory portions and ducts (Fig 1A). To better understand the 3D anatomy of their structures by whole-mount staining, the cross-sectional anatomy was assessed histologically using sweat gland cell markers. HE staining showed that the coiled structures were composed of closely clustered tubules (Fig 1B). These structures were encased in adipose and collagenous connective tissues, and detected in the deep dermis adjacent to the hypodermis, which is consistent with previous histological observations [14]. Each sweat gland compartment was analyzed using markers expressed exclusively by secretory luminal, myoepithelial, ductal luminal, or ductal basal cells. K8, a secretory luminal cell marker [15, 16], was expressed in the luminal layers, but not in the myoepithelial layers of sweat glands (Fig 1C). αSMA, a myoepithelial cell marker [15, 16], was expressed in flattened small cells surrounding the sweat gland myoepithelial layers. K77, a ductal luminal cell marker [17], was expressed in the luminal layers of the sweat gland ducts. S100A2, a ductal basal cell marker [18], was expressed in the sweat gland basal layers. Double immunofluorescence staining was used to verify whether these markers were confined to individual sweat gland compartments (Fig 1D). K8 and αSMA were exclusively detected in the luminal and myoepithelial layers of the same tubules, respectively. Similarly, K77 and S100A2 were distinctively expressed in luminal and basal cells, respectively. Furthermore, K8 and K77 were expressed in the luminal cell layers, and αSMA and S100A2 in the basal cell layers of different tubules. K77-positive lumens were narrower than K8-positive lumens, while S100A2-positive cuboidal basal cells were distinguishable from the flattened αSMA-positive myoepithelial cells. These results indicate that K8/αSMA and K77/S100A2 are expressed in sweat gland secretory portions and ducts, respectively, and are specific markers for the four sweat gland compartments.

**Fig 2:**
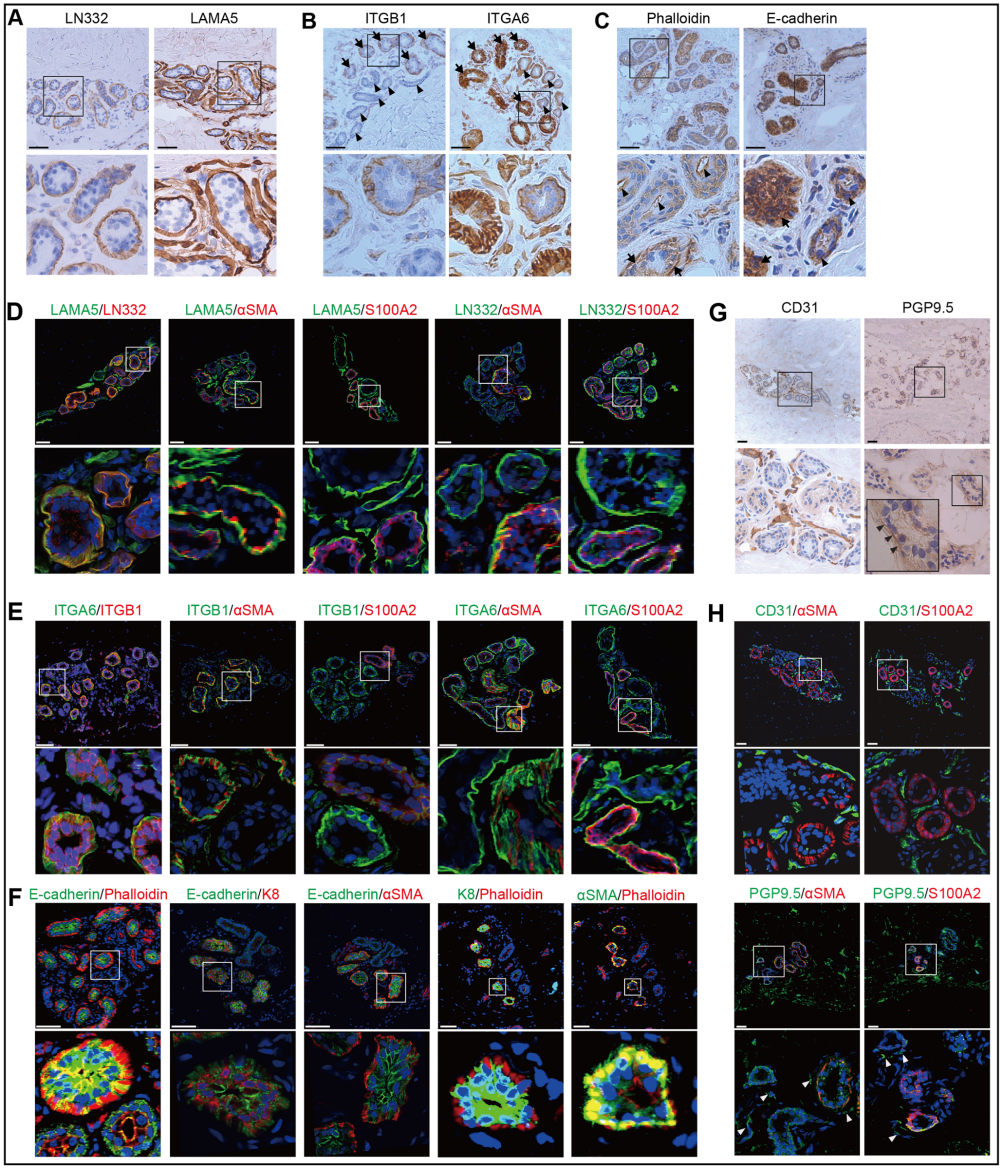
Histological anatomies of basement membranes, cell surface receptors, and cytoskeleton markers in coiled structure compartments of sweat glands. Expression patterns of (A) LN332 and LAMA5 (basement membrane markers), (B) ITGB1 and ITGA6 (cell surface receptor markers), and (C) E-cadherin and phalloidin (cytoskeleton markers) in the basal layers of sweat glands. Arrowheads and arrows in (B) indicate secretory portions and ducts, respectively. Black arrowheads and arrows in (C) indicate strong detection of actin filaments in ductal apical and secretory basal sides, respectively. Arrowheads and arrows indicate expression of E-cadherin in ductal basal and secretory luminal cell layers, respectively. Double immunofluorescence detection of LN332 and LAMA5 (D), and ITGB1 and ITGA6 (E) with sweat gland basal cell markers S100A2 and αSMA, and E-cadherin and phalloidin (F) with sweat gland secretory portion markers K8 and αSMA in sweat gland cross sections. (G) Expression patterns of CD31 (blood vessel) and PGP9.5 (nerve fiber) markers in sweat glands. Sweat glands boxed in the upper panels are shown in the lower panels at higher magnification. Nuclei (blue) were counterstained with Hoechst 33342. Scale bars: 50 μm.

To accurately determine sweat gland cell shapes for subsequent 3D analysis, expression patterns of markers for basement membranes, cell surface receptors, and cytoskeletons were examined in the coiled structure compartments of sweat glands. Laminin-332 (LN332), a major adhesive component of the epidermal basement membrane, was detected in layers surrounding the basal cells of sweat glands (Fig 2A). Laminin α5 (LAMA5), a subunit of laminin-511, was detected on the sweat gland basement membrane and in blood vessels. Integrin β1 (ITGB1) and integrin α6 (ITGA6), both basal epithelial cell surface markers, were expressed in these basal layers, particularly in myoepithelial-like basal cells, indicating their co-expression in myoepithelial cells (Fig 2B). F-actin-based cytoskeletal structures were visualized by staining with phalloidin. Phalloidin was strongly detected in the myoepithelial cell layers, while F-actin filaments had accumulated at the apical side of the ducts (Fig 2C). In contrast, E-cadherin, which is connected to the actin cytoskeleton, was expressed in secretory luminal cells and ductal basal cell layers of sweat glands.

Double immunofluorescence staining of basement membrane markers showed that LAMA5, but not LN332, was preferentially expressed in αSMA-positive, but not S100A2-positive, cell layers, suggesting that LN-511 is predominantly deposited in the secretory portions of sweat glands (Fig 2D). ITGB1 and ITGA6 were co-expressed on the secretory portions and preferentially co-localized with αSMA-positive cell layers (Fig 2E), indicating that basal cells that strongly express ITGB1 and ITGA6 are myoepithelial cells. Phalloidin and E-cadherin were exclusively detected in sweat glands (Fig 2F). In the secretory portions, E-cadherin was co-expressed with K8, but not with αSMA, while phalloidin was detected with αSMA, but not with K8.

Finally, sectional anatomy of the blood vessels and nerve fibers surrounding sweat glands was histologically assessed using CD31 and PGP9.5, specific markers for blood vessels and nerve fibers, respectively. Blood vessels that support sweat reabsorption encased sweat glands, while nerve fibers, which are involved in sympathetic innervation, contiguously resided on the outside of sweat glands (Fig 2G). Double immunofluorescence staining of markers for blood vessels or nerve fibers with those for ducts or secretory portions revealed that blood vessels were biased to the area adjacent to the sweat gland tubules (Fig 2H). Nerve fibers were also found proximal to the sweat gland tubules. Thus, conventional histological analysis showed that blood vessels and nerve fibers were located close to the sweat gland tubules.

### Imaging of the 3D structure of sweat glands

**Fig 3:**
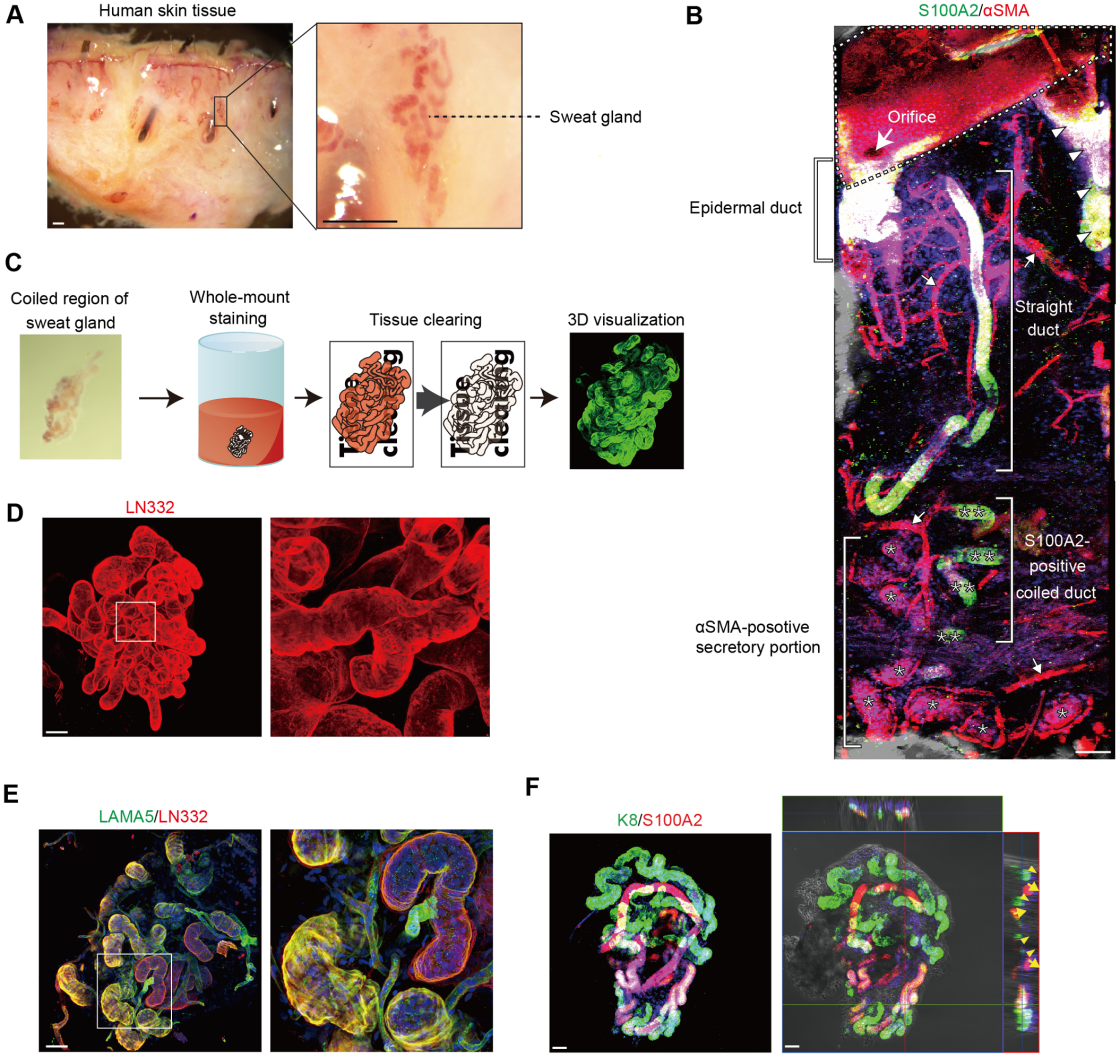
Imaging of the 3D structure of sweat glands. (A) Neutral red-positive sweat gland coiled organs detected in human skin tissue. The boxed region is shown at higher magnification in the left panel. (B) Visualization by whole-mount staining of an entire sweat gland embedded in human skin. αSMA and S100A2 were detected in sweat glands by double immunofluorescence. Asterisks and double asterisks indicate αSMA-positive secretory portions and S100A2-positive coiled ducts, respectively. Arrowheads and arrows indicate hair follicles and blood vessels, respectively. The dashed line indicates the skin surface. (C) Procedure for whole-mount staining of coiled fragments of sweat glands. The left panel shows neutral red-stained sweat gland organs collected from human skin tissue. (D) Visualization of the basement membrane of the entire sweat gland coiled structure by whole-mount staining for LN332. (E) Double immunofluorescence detection of LN332 and LAMA5 in sweat glands. (F) Imaging of ductal and secretory portions of coiled fragments dissected from sweat glands. Double immunofluorescence detection of K8 and S100A2 in an isolated sweat gland. (Left panel) Projection image of a whole-mount 3D sweat gland stained for K8 and S100A2. (Right panel) Optical section of the whole-mount image. Arrows and arrowheads indicate S100A2-positive ductal and K8-positive secretory portions, respectively. Nuclei (blue) were counterstained with Hoechst 33342 (B, E, and F). Boxed areas in the left panels are shown at higher magnification in the right panels (D, E). Scale bars: 200 μm (A) and 50 μm (B, D–F).

Because conventional immunohistochemistry is not sufficient to precisely determine the coiled structures of sweat glands, their detailed anatomy was determined by whole-mount immunostaining, a method that has been used to visualize 3D structures of mammary glands [12]. Sweat glands are very small organs scattered throughout the skin. Therefore, these glands need to be distinguished from the surrounding dermal connective tissue for whole-mount staining. Sweat gland organs were transiently labeled with neutral red, a dye that distinctively stains sweat glands [13], thereby distinguishing them from the dermal connective tissue (Fig 3A). The 3D coiled structures of the neutral red-stained sweat glands were then visualized by whole-mount staining. Skin fragments containing whole sweat glands were immunostained for S100A2 and αSMA, markers for ducts and secretory portions, respectively. Spiral, straight, and coiled ducts expressing S100A2 were detected in the epidermis, upper dermis, and deep dermis, respectively (Fig 3B). The coiled ducts and secretory portions expressing S100A2 and αSMA, respectively, were partially detected in the skin fragments. Because the coiled regions of sweat glands are encased in adipose tissue, resulting in a significant loss of fluorescent signals, the coiled fragments were microsurgically separated using forceps and three-dimensionally visualized by whole-mount staining (Fig 3C). This approach was successful in visualizing the entire coiled structures of sweat glands with LN332-positive tubules detected within the coiled structures that were compactly formed and frequently folded (Fig 3D). Whole-mount staining of sweat glands allowed visualization of bona fide 3D coiled structures. Double immunofluorescence staining of the coiled sweat glands for LAMA5 and LN332 revealed two types of tubules with distinct staining patterns (Fig 3E). One type of tubules predominantly expressed LN332 and was outlined with smooth and cylindrical shapes, while the other type expressed LAMA5 and had an uneven and twisted shape. Interestingly, bundles of actin filament-like structures were recognized on the tubules expressing LAMA5, suggesting that these bundles are the cytoskeleton of myoepithelial cells surrounding secretory portions. These results indicated that LN332 was mostly positive in the coiled sweat duct, but LAMA5 was more specific for the secretory portion. Furthermore, whole-mount analyses revealed differences in 3D coiled structures of ducts and secretory portions. The S100A2-positive ductal tubules were spatially intertwined throughout the coiled regions and embraced in secretory tubules. Notably, K8-positive secretory tubules of coiled regions were frequently bent and formed a self-entangled coiled structure (Fig 3F). Thus, whole-mount staining of sweat glands revealed the entwined and entangled structures of the coiled region of sweat glands, a finding not obtained by conventional histological analyses.

### 3D imaging of a sweat gland at single cell resolution

**Fig 4:**
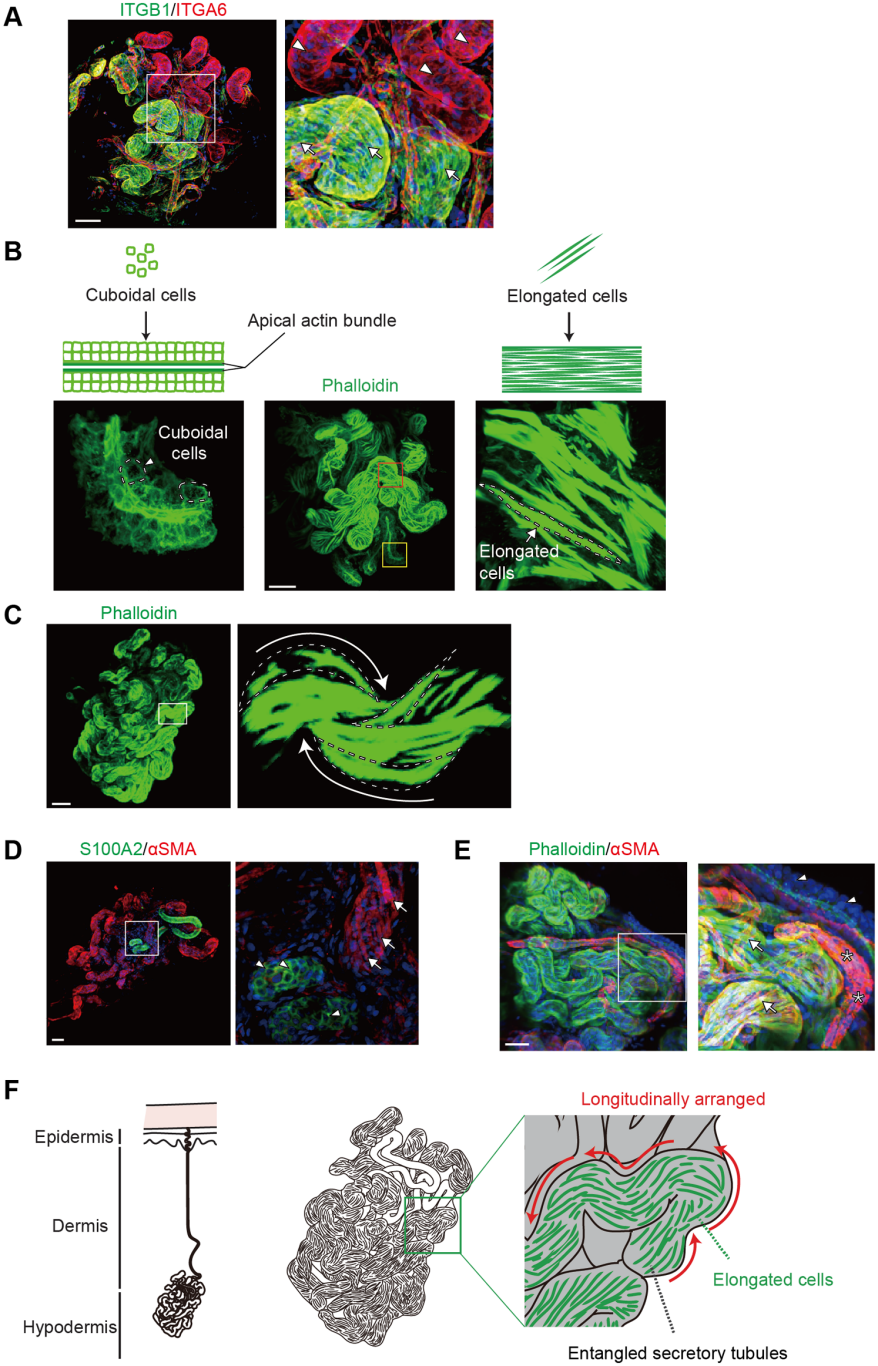
3D imaging of a sweat gland at single cell resolution. (A) Whole-mount 3D confocal images of ITGB1 and ITGA6 expression. Arrowhead and arrow indicate the portions comprised of cuboidal basal cells and elongated cells, respectively. (B) Cuboidal cells (left) and highly elongated cells (right) forming sweat gland putative ducts and secretory portions, respectively. The patterns of F-actin-phalloidin complexes were used to distinguish ducts and secretory portions of sweat glands with the former consisting of cuboidal cells (left) and the latter of highly elongated cells (right). Arrowheads and arrows indicate cuboidal and highly elongated cells, respectively. Yellow and red boxed areas in the center panel are magnified in the left and right panels, respectively. (C) Sweat gland stained with phalloidin. Arrows indicate the elongated cells (dashed lines) that were longitudinally and uniformly arranged on the secretory portion. (D) Whole-mount 3D confocal images of S100A2 and αSMA expression. Arrowhead and arrow indicate the portions comprised of cuboidal basal cells and elongated cells, respectively. (E) Double immunofluorescence detection of αSMA and phalloidin in sweat glands. The right panel shows a high magnification view of the left panel. Arrows indicate phalloidin-positive elongated myoepithelial cells expressing αSMA. Arrowheads indicate ducts. Asterisks indicate αSMA-positive blood vessels. (F) Schematic presentation of the 3D coiled structure of sweat glands. Highly elongated myoepithelial cells are longitudinally arranged on the entangled secretory portion of sweat glands. Boxed areas in the left panels are shown at higher magnification in the right panels (A, C–E). Nuclei (blue) were counterstained with Hoechst 33342 (B, D, and E). Scale bars: 50 μm.

Because the structures of three-dimensionally complex organs are largely determined by cell shapes and arrangements [19], the shape and arrangements of the sweat gland coiled structures were visualized at single cell resolution using cell surface markers ITGB1 and ITGA6. Whole-mount staining showed that ITGB1 and ITGA6 co-localized in the sweat gland basal layers (Fig 4A). The expression patterns of these markers distinguished two types of coiled structures, one composed of cuboidal cells (*arrowheads*) and the other composed of elongated basal cells (*arrows*). To assess cellular arrangements in the coiled tubular structures in greater detail, their F-actin-based cytoskeletal structures were visualized at single cell resolution by fluorescent phalloidin staining. The coiled tubular structures were found to be composed of two portions (Fig 4B), one consisting of cuboidal cells (*arrowheads*), in which actin filaments had accumulated at the apical regions, and the other composed of highly elongated cells that were strongly positive for F-actin-phalloidin complexes (*arrows*). Intriguingly, the elongated cells were longitudinally and uniformly arranged on the secretory portion (Fig 4C). Furthermore, the orientation of the elongated cells was in agreement with the entangled direction of the secretory portion, suggesting that myoepithelial cells are aligned along the tubules to facilitate contractions of the secretory portion, thereby helping sweat excretion.

We next assessed whether the highly elongated cells were myoepithelial cells that surrounded the secretory portions, but not the ducts, of sweat glands. Sweat gland compartments were visualized by 3D analyses with phalloidin, αSMA, and S100A2. αSMA and S100A2 were distinctively expressed in elongated and cuboidal cells of each sweat gland tubule (Fig 4D). Furthermore, αSMA was expressed in the highly elongated cells of the entangled secretory portions and co-localized with F-actin-phalloidin complexes (Fig 4E). Taken together, these whole-mount analyses at single cell resolution revealed the 3D cell shapes and arrangements in sweat gland coiled structures. These findings indicate that the self-entangled coiled structures of secretory portions are attributed to the 3D arrangements of elongated myoepithelial cells that surround the secretory portions (Fig 4F).

### Distributions of sweat gland blood vessels and nerve fibers

**Fig 5:**
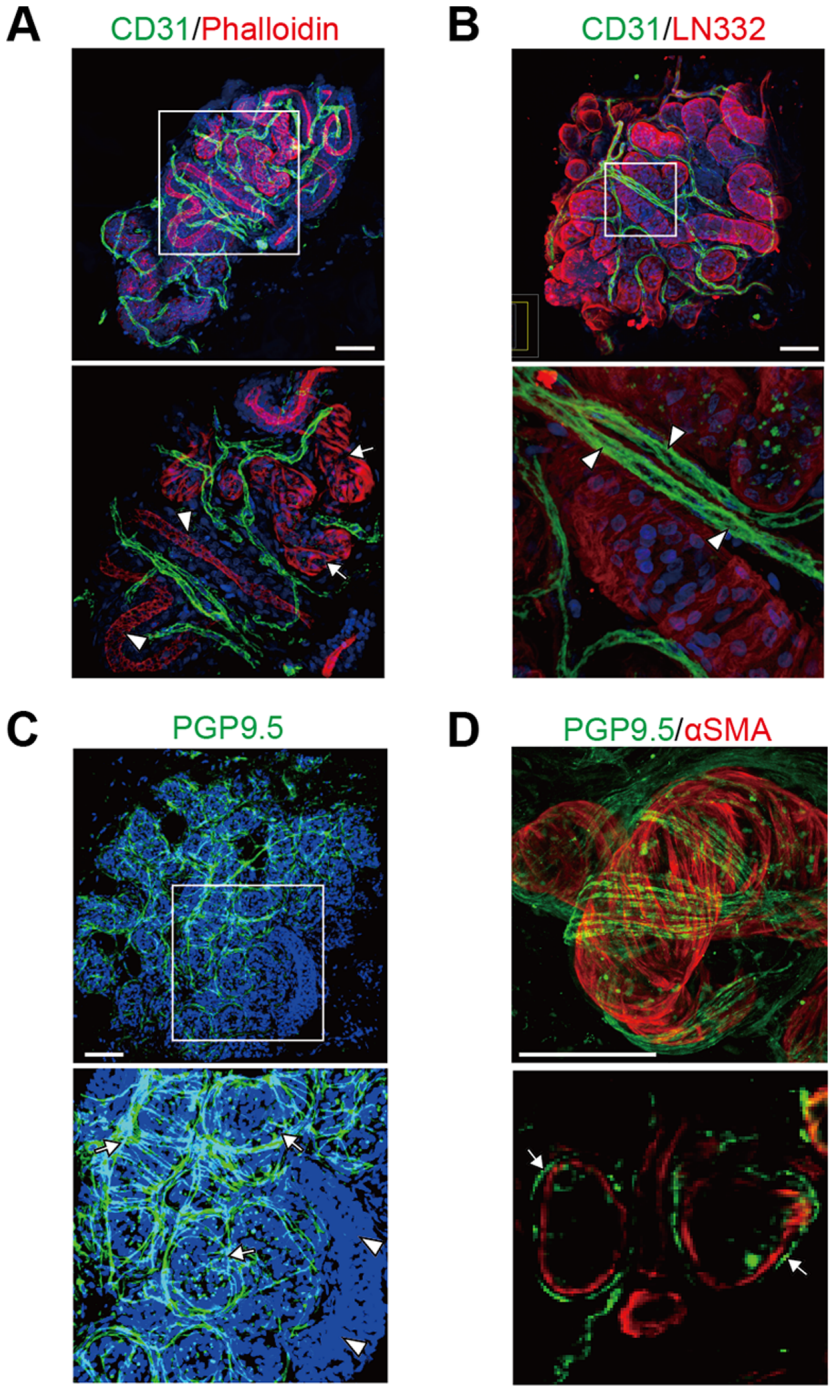
Imaging of sweat gland blood vessels and nerve fibers. (A) Immunofluorescence detection of CD31 and phalloidin in sweat glands. Arrows and arrowheads indicate secretory portions and ducts of sweat gland tubules, respectively. (B) Double immunofluorescence detection of CD31 and LN332 in sweat glands. Arrowheads indicate blood vessels running parallel to sweat gland tubules. (C) Immunofluorescence detection of PGP9.5 in sweat glands. Arrows indicate nerve fibers wrapping around the tubules of sweat glands. Arrowheads indicate ductal tubules. (D) Immunofluorescence detection of PGP9.5 and αSMA in sweat glands. Lower panel shows optical sections of sweat glands. Arrows indicate nerve fibers that enwrap the αSMA-positive secretory portions. The boxed area in the upper panel is shown at higher magnification in the lower panel (A–C). Nuclei (blue) were counterstained with Hoechst 33342. Scale bars: 50 μm.

Considering that blood vessels support the reabsorption of sweat while nerve fibers are involved in sympathetic innervation, understanding the detailed 3D anatomy of blood vessels and nerve fibers surrounding sweat glands may provide insights into the mechanisms that govern sweat gland activity. Immunostaining for the blood vessel marker CD31 revealed that blood vessels were three-dimensionally intertwined with both secretory portions and ducts of sweat glands (Fig 5A) and ran longitudinally in parallel with sweat gland tubules (Fig 5B). Blood vessels in proximity to the ducts are thought to facilitate reabsorption of sweat components [20]. Whole-mount staining also showed that these blood vessels were situated adjacent to not only the ducts, but also the secretory portions of sweat glands, suggesting that blood vessels running along sweat gland tubules are involved in the supply and reabsorption of sweat.

Nerve fibers surrounding sweat glands were visualized by the neuronal marker PGP9.5. PGP9.5-positive nerve fibers were wrapped around the tubules of sweat glands (Fig 5C). Double immunofluorescence staining for PGP9.5 and αSMA showed that these nerve fibers predominantly surrounded the secretory portions of sweat glands (Fig 5D). Furthermore, the nerve fibers covered the flattened myoepithelial cell layers that surrounded the secretory portions, suggesting that the myoepithelial cells respond to sympathetic stimuli from nerve fibers.

## Discussion

**Fig 6:**
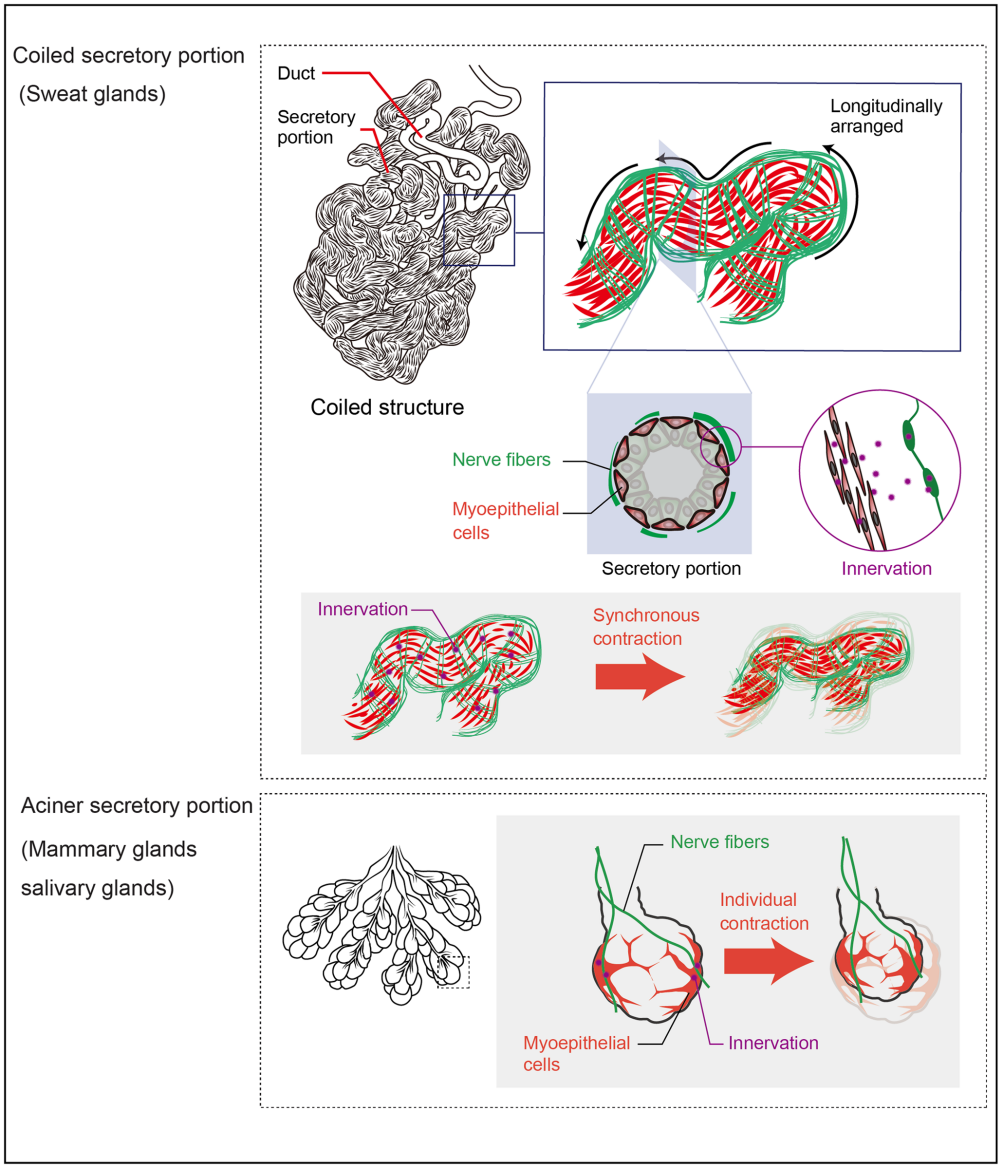
Schematic model for mechanical contraction of sweat glands during sweat secretion. Tubular secretory portions, but not ducts, of sweat glands have self-entangled coiled structural features. Elongated myoepithelial cells are arranged longitudinally parallel to the entangled secretory tubules. Nerve fibers enwrap myoepithelial cell layers surrounding the secretory portions. In sweat glands, multiple elongated myoepithelial cells synchronously contract their tubular secretory portions for excretion of sweat (upper panel), while stellate-shaped myoepithelial cells individually contract their acinar secretory portions of salivary and mammary glands (lower panel).

Although the complex coiled structures of sweat glands have been investigated by 3D reconstruction of serial histological sections, anatomical information is often limited owing to processing losses between sections. This study employed a whole-mount staining method to determine the detailed 3D anatomy of sweat gland coiled structures, which is similar to the approach used for seamless visualization of the 3D structures of mammary glands [12]. Whole-mount staining of sweat glands allowed visualization of bona fide 3D coiled structures that are compactly formed and frequently folded (Fig 6). Furthermore, the method revealed differences in 3D structures of the ducts and secretory portions of these glands. Ducts are occasionally bent and simply folded, while secretory portions are frequently bent and form self-entangled coiled structures. It should be noted that the ducts of coiled regions are embraced in secretory portions, as suggested in an early study from the 1950s [20]. Although the secretory portions of mammary and salivary glands were previously found to have round cyst-like structures, no glands have been shown to have secretory portions with such self-entangled coiled structures [12]. These structural differences among exocrine gland secretory portions suggest that the tubular secretory portions of sweat glands possess three-dimensionally self-entangled coiled structures for effective sweat secretion. Thus, 3D visualization of sweat glands by whole-mount staining has revealed the anatomical features of these coiled structures, which have not been determined by conventional histological methods.

Whole-mount staining for cytoskeletal and cell surface markers succeeded in visualizing the 3D cell shapes and arrangements of each sweat gland compartment. Cuboidal cells are regularly arranged in the ductal basal layers, differing from those in mammary glands, which consist of spindle-shaped myoepithelial cells longitudinally surrounding the mammary gland ducts [12]. Differences in ductal basal cell shapes and arrangements between sweat and mammary glands suggest that sweat gland ducts do not possess the contractile ability to expel fluid. Highly elongated secretory basal cells, including myoepithelial cells, are arranged longitudinally parallel to the entangled secretory tubules. These results indicate that the self-entangled coiled tubular structures may reflect the shape and arrangement of elongated myoepithelial cells on the secretory portions in striking contrast to those of mammary and salivary myoepithelial cells, which are stellate-shaped and scattered throughout the acinar secretory portions [12]. These distinctions suggest that myoepithelial cells primarily define gland-specific shapes and arrangements to effectively induce the contraction of individual 3D structures of exocrine gland secretory portions (Fig 6). Thus, whole-mount analyses of sweat glands at single cell resolution has provided insights into the 3D mechanical contraction of the coiled structures in sweat secretion.

Whole-mount analyses not only revealed 3D arrangements of the coiled structures of sweat glands, but also those of the blood vessels and nerve fibers that facilitate sweat gland activity. Although blood vessels and nerve fibers are abundant in sweat glands, their 3D arrangements have been difficult to determine by conventional histological methods. The whole-mount analyses presented here revealed that the blood vessels run longitudinally parallel to the tubular structures of sweat glands. Intriguingly, the blood vessels are consistently close to, but separated from, the sweat gland tubules in a parallel arrangement similar to that of peritubular capillaries and proximal renal tubules in the kidney [21]. In the kidney, the peritubular capillaries are distributed in close proximity to the proximal renal tubules and reabsorb their filtrates. Thus, the adjacent arrangement of blood vessels and sweat glands may facilitate effective provision and reabsorption of sweat components. Whole-mount analyses also revealed the 3D distribution of the nerve fibers that enwrap the myoepithelial cell layers surrounding the secretory portions. However, in salivary exocrine glands, the nerve fibers extend to, but do not wrap, the stellate-shaped myoepithelial cells on secretory acini [4, 5]. The difference between sweat and salivary glands in terms of the spatial arrangement of nerve fibers relative to myoepithelial cells suggests that the myoepithelial cells of salivary glands individually contract their secretory acini, while the multiple myoepithelial cells of sweat glands synchronously contract their secretory portions (Fig 6).

There is accumulating evidence that sweat glands contain stem cells with a regenerative potential. Lu et al. [22] demonstrated that mouse sweat glands harbor stem cells in sweat ducts and secretory coils, which differentially respond to injuries and exhibit distinct regenerative potentials. Rittie et al. reported that duct cells in human sweat glands contribute to re-epithelialization of the skin after trauma [23]. We have previously shown that myoepithelial cells are enriched with sweat gland stem cells possessing the ability to regenerate sweat gland-like spheres with apical and basal cell layers that express luminal (K8) and myoepithelial (αSMA) markers, respectively [24, 25]. We also revealed that myoepithelial cells including stem cells are closely surrounded by neighboring nerve fibers, indicating that sweat gland stem cells reside in a unique environment. Stem cells of various tissues reside within unique microenvironments called niches [26, 27]. These niches consist of microenvironmental cells that nurture stem cells and enable them to maintain tissue homeostasis [28, 29]. Nerve cells are also known to comprise the cellular components of the niche. Nerve fibers that wrap the upper bulge regions of hair follicles secrete sonic hedgehog, a signaling molecule vital for neural development, and create a molecularly and phenotypically distinct hair follicle stem cell population [30]. In salivary glands, parasympathetic innervation occurs in parallel with salivary gland development [31] and is essential for regeneration after injury [32]. Furthermore, parasympathetic innervation has been shown to maintain the salivary gland progenitor population in an undifferentiated state, which is required for salivary organogenesis [4]. Although sweat glands are innervated by sympathetic nerves, it remains unknown whether these nerves are part of the niches that maintain sweat gland stem cells. We found that nerve fibers surround the sweat gland secretory portions, but they hardly surround the ducts. Myoepithelial cells respond to neurotransmitters secreted by sympathetic nerves and induce contraction of glandular tubules for sweat secretion, suggesting that the sweat gland stem cells among the myoepithelial cells adjacent to the sympathetic nerves may be largely influenced by their neural microenvironment.

Sweat glands encompassed by nerve fibers have cholinergic sympathetic innervation [2] and excrete sweat upon stimulation by acetylcholine secreted from nerve fibers. Neurological disorders that affect the central or peripheral nervous systems lead to sweating disorders classified by the amount of secreted sweat, i.e., anhidrosis, hypohidrosis, and hyperhidrosis [33]. We quantified nerve fiber densities around 3D sweat gland structures using the method of Gibbons et al [34]. The nerve fiber densities obtained in this study were slightly higher than those reported by Gibbons et al (S1 Fig), mostly because of the high resolution detection of nerve fibers by our 3D imaging techniques. The improved quantification of nerve fiber densities in sweat glands may contribute to the assessment of sudomoter functions in neuropathy patients including those with diabetes.

Collectively, our whole-mount analyses revealed the complex coiled structures of human sweat glands at single cell resolution. Sweat glands possess three-dimensionally self-entangled coiled secretory portions, and the entangled orientation of these secretory portions is consistent with the 3D arrangement of myoepithelial cells. Furthermore, these myoepithelial cells are closely covered by nerve fibers, indicating that myoepithelial cells effectively contract the secretory portions following innervations. Thus, the 3D anatomical information obtained by whole-mount staining of sweat glands provides insights to better understand the mechanisms of sweat secretion.

## Supporting information

S1 Fig Nerve fiber densities in 3D sweat gland structures. Sweat gland nerve fiber densities were quantified according to Gibbons et al (2009) [34] with slight modification. (A) 3D objects of nerve fibers and sweat glands were individually extracted using Imaris software (Andor Technology Ltd, Belfast, UK). (B) The extracted 3D object with the selected area of interest is highlighted by the red dashed line. (C) Nerve fibers in the extracted image (B) are overlaid with a grid. Any circle partially or wholly contained within the area of interest is eligible for counting. (D) Nerve fibers that intercept the grid are counted manually. Nerve fibers that touch, but do not enter, the circle are not counted. (E) The nerve fiber density in the sweat gland is shown by the gray box plot (*N* = 6). The box plot demonstrates the median value with the first and third quartiles outlined by the box, 0^th^ and 100^th^ percentiles by the whisker lines, and individual values shown as solid dots. Scale bars: 50 μm (A–D).
(TIF)
