# Supplementary figures and images for: Three-dimensional cell shapes and arrangements in human sweat glands as revealed by whole-mount immunostaining
Source: PLoS One. 2017 Jun 21;12(6):e0178709. doi: 10.1371/journal.pone.0178709 (PMC5479532; doi:10.1371/journal.pone.0178709)

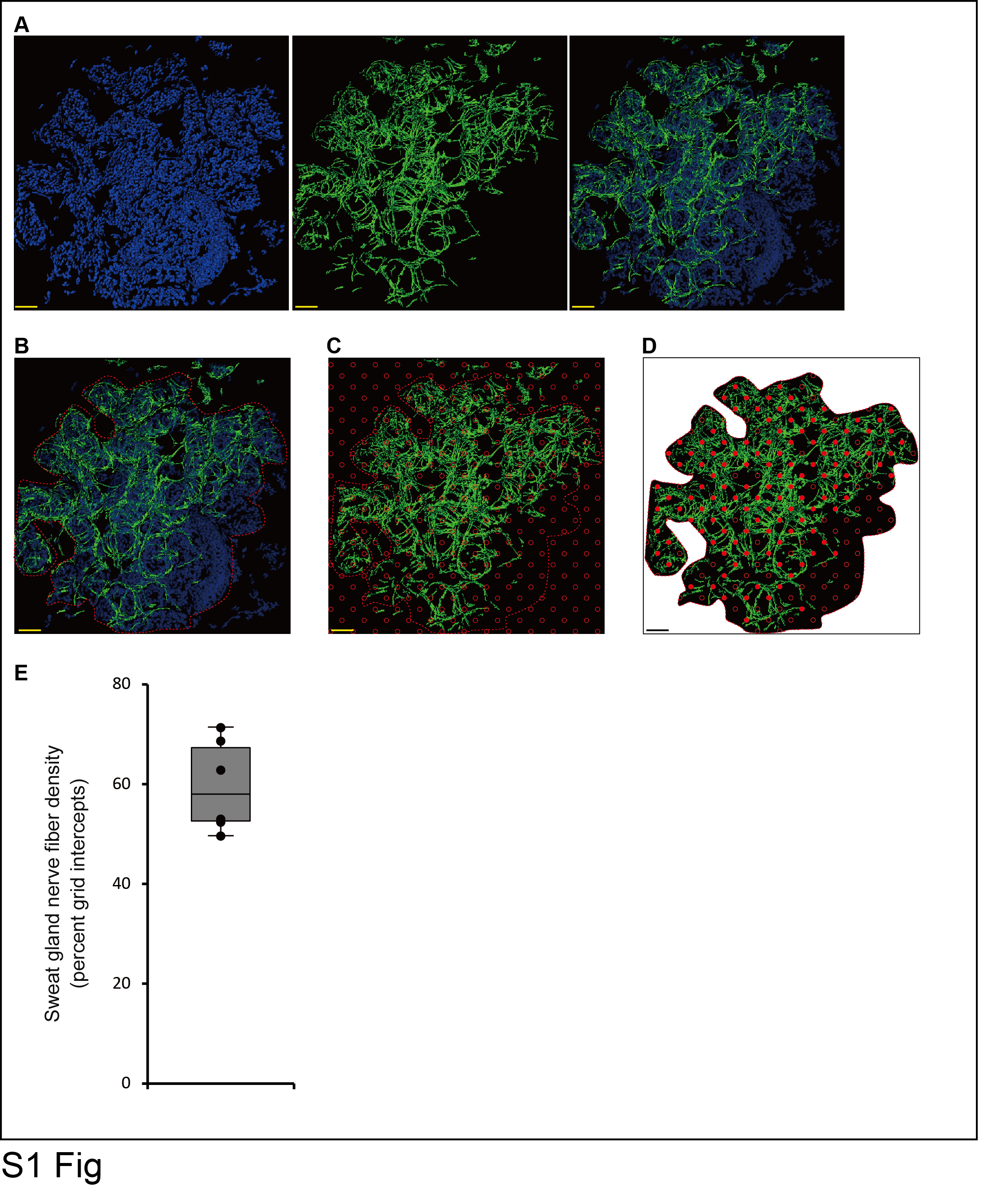

Supplement: S1 Fig — Sweat gland nerve fiber densities were quantified according to Gibbons et al (2009) [34] with slight modification. (A) 3D objects of nerve fibers and sweat glands were individually extracted using Imaris software (Andor Technology Ltd, Belfast, UK). (B) The extracted 3D object with the selected area of interest is highlighted by the red dashed line. (C) Nerve fibers in the extracted image (B) are overlaid with a grid. Any circle partially or wholly contained within the area of interest is eligible for counting. (D) Nerve fibers that intercept the grid are counted manually. Nerve fibers that touch, but do not enter, the circle are not counted. (E) The nerve fiber density in the sweat gland is shown by the gray box plot (N = 6). The box plot demonstrates the median value with the first and third quartiles outlined by the box, 0th and 100th percentiles by the whisker lines, and individual values shown as solid dots. Scale bars: 50 μm (A–D). (TIF) [file pone.0178709.s001.tif]
